# Association of experienced and evaluative well-being with health in nine countries with different income levels: a cross-sectional study

**DOI:** 10.1186/s12992-017-0290-0

**Published:** 2017-08-23

**Authors:** Marta Miret, Francisco Félix Caballero, Beatriz Olaya, Seppo Koskinen, Nirmala Naidoo, Beata Tobiasz-Adamczyk, Matilde Leonardi, Josep Maria Haro, Somnath Chatterji, José Luis Ayuso-Mateos

**Affiliations:** 1 Department of Psychiatry, Universidad Autónoma de Madrid, Arzobispo Morcillo 4, 28029 Madrid, Spain; 2 Instituto de Salud Carlos III, Centro de Investigación Biomédica en Red de Salud Mental. CIBERSAM, Madrid, Spain; 3 Department of Psychiatry, Hospital Universitario de La Princesa, Instituto de Investigación Sanitaria Princesa (IIS-Princesa), Madrid, Spain; 4 Parc Sanitari Sant Joan de Déu, Universitat de Barcelona, Sant Boi de Llobregat, Barcelona, Spain; 5 National Institute for Health and Welfare, Helsinki, Finland; 6 Department of Health Statistics and Information Systems, World Health Organization, Geneva, Switzerland; 7 Department of Medical Sociology, Jagiellonian University Medical College, Krakow, Poland; 8 Fondazione IRCCS, Neurological Institute Carlo Besta, Milan, Italy

**Keywords:** Experienced well-being, Evaluative well-being, Health status

## Abstract

**Background:**

It is important to know whether the relationships between experienced and evaluative well-being and health are consistent across countries with different income levels. This would allow to confirm whether the evidence found in high income countries is the same as in low- and middle-income countries and to suggest policy recommendations that are generalisable across countries. We assessed the association of well-being with health status; analysed the differential relationship that positive affect, negative affect, and evaluative well-being have with health status; and examined whether these relationships are similar across countries.

**Methods:**

In this cross-sectional study, interviews were conducted amongst 53,269 adults from nine countries in Africa, Asia, Europe, and Latin America. Evaluative well-being was measured with a short version of the World Health Organization (WHO) Quality of Life instrument, and experienced well-being was measured with the Day Reconstruction Method. Decrements in health were assessed with the 12-item version of WHO Disability Assessment Schedule 2.0. Block-wise linear regression and structural equation models were employed.

**Results:**

Considering the overall sample, evaluative well-being was more strongly associated with health (β = −0.35) than experienced well-being (β = −0.14), and negative affect was more strongly associated with health (β = 0.10) than positive affect (β = −0.02). The relationship between health and well-being was similar across countries. Lower scores in evaluative well-being and a higher age were the factors more strongly related with a worse health.

**Conclusions:**

The different patterns observed across countries may be related to differences in the countries’ gross domestic product, social protection system, economic situation, health care provision, lifestyle behaviours, or living conditions. The fact that evaluative well-being is more predictive of health than experienced well-being suggests that our level of satisfaction with our lives might be more important for our health than the actual emotions than we experience in our day-to-day lives and points out the need of interventions that improve the way people evaluate their lives.

## Background

Well-being is a priority on the global public health agenda. The general goal of the World Health Organization (WHO) Mental Health Action Plan 2013–2020 is to promote well-being and prevent mental disorders. The Plan states that governments should put in place actions to protect and promote well-being at all stages of life [1]. Moreover, the High-Level Panel of Eminent Persons on the Post-2015 Development Agenda has acknowledged the importance of ensuring that every person achieves a basic standard of well-being [2].

Two different components of well-being can be distinguished. Evaluative well-being captures the global evaluation that people make about their life, which is usually assessed by reporting how satisfied people are with their lives whereas experienced well-being refers to the positive and negative emotions that people experience day-to-day [3, 4]. The literature on well-being also establishes a third component of well-being, the eudaimonic one [5], which is focused on self realisation, sense of purpose, and meaning in life.

There is a bidirectional relationship between well-being and health. Health is strongly correlated with both experienced and evaluative well-being, even after controlling for history of depression, age, income and other sociodemographic variables [4]. In turn, well-being might also be a protective factor for health, reducing the risk of chronic illness and promoting longevity [6, 7].

People with high evaluative well-being experience less pain [8] and self-report better health [9, 10]. Experienced well-being might also have an impact on health [10]. Greater enjoyment of life has been found to be associated with reduced risk of developing impaired activities of daily living and with a slower decline in gait speed [11]. Meta-analysis of prospective studies found that experienced positive affect [12, 13] and evaluative well-being [13] were associated with reduced mortality in healthy populations. In the United Kingdom, it was found that high experienced positive affect was associated with longer survival, after controlling for baseline health status and other covariates, whereas negative affect and depressed mood were not associated with survival after controlling for covariates [14]. In Spain experienced positive affect was found to be associated with decreased risk of mortality in people without depression [15]. In contrast, other studies did not find that positive affect predicted a reduced risk of coronary heart disease [16] or subsequent good levels of health [17].

Although the previous evidence shows that well-being might have an impact on health, there is still a need to know which is the differential association that the evaluative and experiential components of well-being have on health, and the differential association of positive and negative affect on health. Furthermore, even though more than 85% of the world’s population lives in low- and middle-income countries, most of the evidence comes from high-income countries, and the evidence from low- and middle-income countries is scarce [6, 18]. There is, therefore, a need to know whether these relationships are consistent across countries with different income levels in order to suggest policy recommendations that are generalisable across countries. This gap in knowledge could be filled by analysing data from the different components of well-being that have been collected from nationally representative samples from multi-continent studies using the same methodology across countries.

The main aim of the present study was to examine the relationship between different components of well-being and health status; specifically, to analyse which of the well-being variables analysed (positive affect, negative affect, and evaluative well-being) showed the strongest association with health. Moreover, it was assessed whether these relationships showed a similar trend across a set of countries with different income levels.

## Methods

### Sample and procedure

The data were obtained from the WHO Study on Global AGEing and Adult Health (SAGE) [19] and the Collaborative Research on Ageing in Europe (COURAGE in Europe) [20]. Nationally representative samples of the adult population (i.e., people aged 18+ years) were obtained for the nine countries considered in both surveys: China, Ghana, India, Mexico, Russia and South Africa, from SAGE; and Finland, Poland, and Spain, from COURAGE in Europe. Beyond that, SAGE and COURAGE in Europe are surveys focused on ageing population, with an emphasis on populations aged 50+ years, and people older than 80 years were overrepresented in the sampling in order to avoid having small sample sizes for the oldest age groups.

The nine countries implemented a multistage cluster sampling design resulting in nationally representative cohorts. Person-level analysis weights, which included sample selection and a post-stratification factor, were calculated for each country. In terms of socio-demographics, post stratification used the most recent estimates provided by the national statistical offices of the respective countries. More specific details about the sample design can be found elsewhere for SAGE [19] and COURAGE in Europe [4].

Both surveys used a similar questionnaire and the health and well-being variables considered in the present study were asked and measured in the same way. Other studies have employed data from both surveys and have analysed them jointly [21–23]. SAGE is a longitudinal study, and the data used in this work corresponds to SAGE Wave 1, conducted between 2007 and 2010. The interviews in COURAGE in Europe project were conducted in 2011 and 2012. Specifically, face-to-face interviews were conducted in China in 2008–2010, in Finland in 2011, in Ghana in 2008–2009, in India in 2007–2008, in Mexico in 2009–2010, in Poland in 2011, in the Russian Federation in 2007–2010, in South Africa in 2007–2008 and in Spain in 2011–2012. Countries included in SAGE (China, Ghana, India, Mexico, the Russian Federation and South Africa) were low- and middle-income countries, whereas the countries included in COURAGE (Finland, Poland and Spain) were high-income countries [24] according to the World Bank Classification, considering the country’s position in the World Bank ranking when the fieldwork in the corresponding country started.

The overall sample comprises 53,269 non-institutionalised adults from China (14,811), Finland (1976), Ghana (5108), India (11,230), Mexico (2742), Poland (4071), Russia (4355), South Africa (4223), and Spain (4753), representing different geographical locations and different levels of socio-economic and demographic transition. Face-to-face interviews were conducted at the respondents’ homes by lay trained interviewers. Quality control procedures were undertaken during the fieldwork [25]. SAGE was approved by the World Health Organization’s Ethical Review Committee. Additionally, partner organisations in each country implementing SAGE [19] obtained ethical approvals through their respective institutional review bodies. Ethical approval for the COURAGE in Europe survey was obtained from the local ethics research review boards [4]. Written informed consent from each participant was also obtained in both studies.

The individual response rate ranged from 53% in Finland and Mexico to 93% in China. If a participant was cognitively impaired and not able to respond to the interview, a shorter version of the interview that did not include items related to well-being among others, was administrated to a proxy respondent. The sample considered for the analyses conducted in the present manuscript comprised those participants who answered the questions about health and well-being in the SAGE and COURAGE in Europe questionnaires. Measures of health and well-being employed in both surveys are described below.

### Measures

Participants were asked to provide sociodemographic information (age, sex, current marital status, educational attainment, household income, and residential setting) at the beginning of the interview.

The 12-item version of WHO Disability Assessment Schedule 2·0 (WHODAS 2·0) [26], a generic assessment instrument developed by the WHO to provide a standardised method for measuring health and disability across cultures, [26] was used to evaluate decrements in health. WHODAS evaluates six domains of day-to-day functioning in the previous 30 days. Scores on the 12 negative items were summed and transformed into a 0–100 scale, with 0 indicating minimum disability/best functional ability and 100 indicating maximum disability/worst functional ability. The WHODAS is an international cross-cultural instrument [27] and its 12 items perform well at discriminating varying levels of disability [28]. In the present study, the 12-item WHODAS 2·0 showed a good internal reliability, with Cronbach’s alpha values ranging from 0.89 in Finland to 0.94 in Poland.

Experienced well-being was assessed using an abbreviated version of the Day Reconstruction Method (DRM) [21] developed and validated for use in large population surveys. The DRM measures affective experiences, reporting seven emotions associated to the activities conducted in an specific time frame of the previous day. Participants are asked to reconstruct a portion (morning, afternoon, or evening) of their previous day’s activities, and report the positive (calm/relaxed and enjoying) and negative (worried, rushed, irritated/angry, depressed, and tense/stressed) emotions associated with each activity. Positive affect and negative affect were defined as the average of the positive and negative emotions, respectively, weighted by the duration of the activities. The scores obtained were transformed into a 0–100 scale, with higher values indicating higher positive and higher negative affect, respectively. The DRM has shown adequate reliability [21, 29], construct validity [21, 30], temporal stability [31] and measurement invariance [29] in previous studies conducted in some of the countries considered in SAGE and COURAGE in Europe surveys.

Evaluative well-being was assessed using a short version of the WHO Quality Of Life (WHOQOL) instrument, which has shown good cross-cultural field study performance and satisfactory convergent and discriminant validity [32]. Throughout the eight items, respondents were asked to rate their overall life satisfaction, energy, money to meet their needs, and their satisfaction with himself/herself, ability to perform daily activities, health, personal relationships, and living place conditions. Conceptually, the WHOQOL items represent psychological, physical, social and environmental domains. A composite score was obtained by a simple sum of scores on the eight items. The final score was transformed into a 0–100 scale, with higher scores indicating better evaluative well-being. In the present study, the instrument showed an adequate internal reliability, with Cronbach’s alpha values ranging from 0.81 in Mexico and 0.86 in China.

The full questionnaire for the SAGE study is available at http://www.who.int/healthinfo/systems/GenericIndividualQ.pdf. All of the questions were translated from English into the local languages, following the WHO translation guidelines for assessment instruments [33].

### Statistical analysis

All data were weighted to account for sampling design in each country and to generalise the study sample to the reference population. Post-stratification corrections were made to the weights to adjust for the population distribution obtained from the national census from each country and for non-response [34]. The Taylor series linearisation method [35], widely used in complex sample designs, was employed to estimate sampling errors.

Hedges’ *g* for unpaired t-tests and Cramer’s *V* for contingency table chi-square tests were reported to assess the effect sizes associated with possible differences in socio-demographics between the final and the excluded sample (i.e. those participants with missing values on the self-reported health questions which comprised the WHODAS or well-being measures, such as proxy participants who were not asked those questions). Values of 0.20, 0.50, and 0.80 for Hedges’ *g* and of 0.10, 0.30, and 0.50 for Cramer’s *V* constitute small, medium, and large effect sizes, respectively. The characteristics of the final sample in each country were described.

The relative contribution of evaluative and experienced well-being to health status was examined by means of a block-wise linear regression analysis conducted over the overall sample. The WHODAS score was considered as the dependent variable. A first block comprising socio-demographic characteristics was included to account for their potential confounder relationship with the WHODAS score. Educational attainment and household income were dichotomised, considering as reference categories an educational level lower than secondary school and being in the first or in the second quintile of income, respectively. Then, the well-being variables were introduced sequentially in two additional blocks. Positive affect and negative affect were jointly included in the second block, since they are two different measures of the experienced well-being component. Finally, evaluative well-being was introduced in a third block and the final model (comprising the three blocks considered) reported.

Dummy variables for each country were also included in the first block, considering China as reference category since it was the country with the largest sample size. These dummy variables were included in the model in order to adjust for the potential confounder effect of country in the relationship between well-being and health, removing potential differences across countries when pooling the data. The increase in the proportion of variance explained in each block, Δ*R* ^2^, was tested at each step by means of the difference in the likelihood ratio chi-square for each model, which tests the null hypothesis that each additional set of predictors contributes nothing beyond the set(s) of variables entered in the model(s) at earlier steps. Confidence intervals (CI) for the coefficients and effect size measures (beta coefficients) were also provided.

A Structural Equation Model (SEM) framework was employed to assess how the experiential and evaluative components of well-being were associated with health. In this case, experienced well-being was a latent variable that was inferred from positive and negative affect, whereas evaluative well-being was measured from the WHOQOL score. Continuous variables were standardised and the sign for negative affect was changed in the model so variables in a same construct had the same direction. Dummy variables for each country and socio-demographic covariates with a *p <* 0.001 in the previous regression model were included. The maximum likelihood estimator with robust standard errors (MLR) was used, computing standard errors by means of a sandwich estimator; the MLR estimator uses robust standard errors [36] and is robust to non-normality. The standardised coefficients were also reported, and can be interpreted as effect size measures.

Root Mean Square Error of Approximation (RMSEA) and Standardised Root Mean Square Residual (SRMR) were employed to assess fit of the SEM model conducted over the pooled sample, according to the cut-off values (RMSEA < 0.08; SRME < 0.08) proposed in the literature for SEM [37, 38]. Finally, in order to check whether there was a similar pattern in the relationship between well-being components (experienced and evaluative well-being) and health across countries with different income levels, a structural equation model was run separately for each country.

Mplus version 6 was used for structural equation modelling and Stata SE version 11 for the remaining analyses. CI were constructed at the 95% confidence level.

## Results

Interviews were conducted with 53,269 people from China, Finland, Ghana, India, Mexico, Poland, Russia, South Africa, and Spain. However, 1926 of these (3.6% of the initial participants) were removed from the analyses because they did not answer the health and well-being sections analysed in the present article. The differences between respondents excluded and included were significant, but they had associated a small effect size, suggesting that they are probably due to the large overall sample size. These differences break down as follows: sex, 57.0% women in the final sample vs. 59.5% women in the sample removed (Cramer’s *V* = 0.01); mean age, 58.0 ± 15.1 years vs. 60.7 ± 17.6 years (Hedges’ *g* = 0.17); percentage of people married or in partnership, 69.7% vs. 68.7% (Cramer’s *V* = 0.01); and percentage of people living in a rural setting, 47.3% vs. 49.9% (Cramer’s *V* = 0·01).

The socio-demographics corresponding to the final sample, broken down by country, are shown in Table 1. The percentage of women in the sample ranged from 47.3% in Ghana to 64.7% in Russia, while the mean age ranged from 50.0 ± 16.6 years in India to 63.1 ± 14.0 in Mexico. The highest percentages of people who had completed secondary school or higher level were found in Russia (90.2%), Finland (87.9%), and Poland (75.0%). The percentage of people married or in partnership was higher in China (83.7%) and India (77.6%), whereas the percentage of people living in a rural setting ranged from 13.6% in Spain to 74.7% in India.

**Table 1:** Socio-demographic characteristics and mean estimates (95% CI) on WHODAS and well-being scores, by country

	China (*n* = 14,235)	Finland (*n* = 1855)	Ghana (*n* = 4889)	India (*n* = 11,203)	Mexico (*n* = 2629)	Poland (*n* = 3929)	Russia (*n* = 4165)	South Africa (*n* = 3855)	Spain (*n* = 4583)
Socio-demographics									
Female: *n* (%)	7619 (53.5)	1052 (56.7)	2314 (47.3)	6866 (61.3)	1625 (61.8)	2360 (60.1)	2694 (64.7)	2219 (57.6)	2505 (54.7)
Age, years: Mean ± s.d.	60.33 ± 11.85	58.41 ± 15.99	60.14 ± 14.03	50.02 ± 16.60	63.14 ± 13.97	57.02 ± 17.93	62.35 ± 13.01	60.33 ± 12.28	59.71 ± 15.90
Secondary school or higher level completed: *n* (%)	5852 (41.1)	1629 (87.9)	1286 (26.3)	3248 (29.0)	618 (23.5)	2945 (75.0)	3755 (90.2)	944 (24.5)	2048 (44.7)
Married or in partnership: *n* (%)	11,908 (83.7)	1174 (63.3)	2914 (60.0)	8695 (77.6)	1670 (63.5)	2194 (55.8)	2370 (57.0)	1996 (52.8)	2777 (60.6)
Rural setting: *n* (%)	7318 (51.4)	414 (22.3)	2871 (58.7)	8364 (74.7)	699 (26.6)	1698 (43.2)	1023 (24.6)	1270 (33.0)	625 (13.6)
Mean estimates (95% CI)									
WHODAS	3.66 (3.43,3.90)	6.38 (5.84,6.93)	12.39 (11.47,13.31)	16.39 (15.91,16.87)	8.95 (7.35,10.56)	11.02 (10.30,11.74)	14.07 (12.97,15.17)	9.60 (7.91,11.29)	6.69 (6.21,7.17)
Positive affect	73.54 (72.29,74.79)	77.24 (75.93,78.55)	79.84 (78.46,81.23)	62.61 (61.73,63.48)	66.80 (63.82,69.79)	72.34 (70.65,74.03)	64.82 (63.00,66.64)	85.95 (83.08,88.81)	80.99 (80.16,81.83)
Negative affect	2.70 (2.32,3.08)	6.85 (6.15,7.54)	4.56 (3.97,5.14)	11.27 (10.80,11.75)	11.32 (9.52,13.13)	7·91 (7.16,8.66)	10.84 (9.74,11.95)	4.58 (3.14,6.02)	11.16 (10.49,11.83)
Evaluative well-being	68.08 (67.52,68.64)	78.27 (77.49,79.06)	64.73 (63.80,65.65)	66.79 (66.33,67.26)	67.47 (65.65,69.28)	69.72 (68.78,70.66)	64.61 (63.69,65.54)	65.49 (63.33,67.65)	75.31 (74.65,75.96)

Unweighted data for socio-demographics; weighted data were used to obtain mean estimates on WHODAS and well-being scores. WHODAS, positive affect, negative affect and evaluative well-being scores ranged between 0 and 100. Higher scores in WHODAS indicate a worse health. Higher scores in positive affect, negative affect and evaluative well-being indicate a higher score in positive affect, negative affect and evaluative well-being, respectively

Mean estimates of WHODAS and well-being scores, by country, are also shown in Table 1. The best health status was found in China, Finland, and Spain, while the worst health status was found in India. The highest positive affect scores were found in South Africa and Spain, whereas the lowest negative affect scores were found in China, Ghana, and South Africa. The three highest scores for evaluative well-being were found in Finland, Spain, and Poland.

The percentage of variance in WHODAS score explained by the final regression model conducted over the overall sample was 47.9% (Table 2). Evaluative well-being and age showed the strongest associations with the WHODAS score. Age was inversely related with health, and so directly related with the WHODAS score (β = 0.34). Evaluative well-being presented a stronger association with the WHODAS score (β = −0.34) than the two components of experienced well-being (β = 0.10 for negative affect and β = −0.02 for positive affect). A higher score for negative affect was associated with worse health; this association was clearly stronger than the association between positive affect and health. People who were married or in partnership (β = −0.08) and people whose educational attainment was secondary school or higher (β = −0.05) showed lower scores on the WHODAS. Finally, being female was associated with worse health (β = −0.06). In summary, a higher score in negative affect was associated with worse health, while higher scores in evaluative well-being and positive affect were related to a better health, after controlling for potential confounders. However, effect sizes associated varied across these well-being variables.

**Table 2:** Final block-wise linear regression model considering WHODAS score as dependent variable

Variables	Coef. (95% CI)	*p*-value	β
Intercept	18.54 (16.49,20.59)	<0.001	−
First block	*R* ^*2*^ = 0.349		
Sex (ref. Female)	−1.87 (−2.32,−1.42)	<0.001	−0.06
Age	0.34 (0.32,0.36)	<0.001	0.34
Married or in partnership (ref. Not married or in partnership)	−2.93 (−3.57,−2.29)	<0.001	−0.08
Education attainment (ref. Lower than secondary school)	−1.53 (−2.08,−0.98)	<0.001	−0.05
Residential setting (ref. Rural)	−0.88 (−1.40,−0.36)	0.002	−0.03
Household income (ref. 1st or 2nd quintile)	0.10 (−0.41,0.62)	0.70	0.01
Country (ref. China)			
Finland	4.67 (3.93,5.41)	<0.001	0.02
Ghana	6.85 (6.04,7.67)	<0.001	0.05
India	11.86 (11.32,12.40)	<0.001	0.37
Mexico	4.38 (2.97,5.79)	<0.001	0.01
Poland	6.77 (6.04,7.49)	<0.001	0.08
Russia	5.21 (4.36,6.06)	<0.001	0.13
South Africa	5.03 (3.70,6.35)	<0.001	0.06
Spain	3.55 (2.87,4.24)	<0.001	0.05
Second block, Δ*R* ^*2*^	*R* ^*2*^ = 0.385; Δ*R* ^*2*^ = 0.036		
Positive affect	−0.02 (−0.03,−0.01)	0.007	−0.02
Negative affect	0.12 (0.10,0.15)	<0.001	0.10
Third block, Δ*R* ^*2*^	*R* ^*2*^ = 0.479; Δ*R* ^*2*^ = 0.094		
Evaluative well-being	−0.37 (−0.39,−0.35)	<0.001	−0.34

Δ*R* ^*2*^ = Change in *R* ^*2*^ regarding the previous model. Dummy variables for each country were included in order to control its potential confounder effect

General analysis conducted over the overall sample. Weighted data

**Fig. 1:**
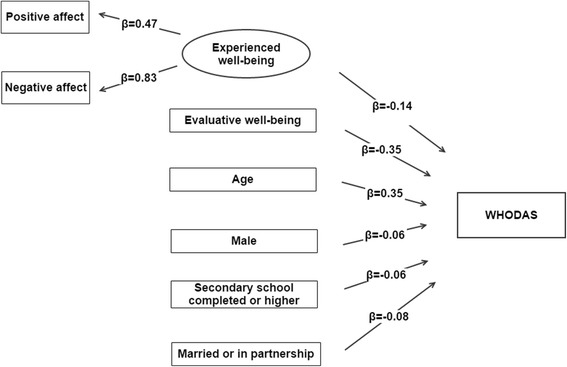
SEM estimates of the association of experienced and evaluative well-being with WHODAS score. Weighted data β=Standardised coefficients; all the coefficients had associated *p* < 0.001. Analyses were controlled by country

Residential setting and household income were not included in the SEM model conducted over the global sample, since the threshold established in *p* = 0.001 was exceeded and a small effect size (|β| ≤ 0.03) was found in the regression model. The results corresponding to the SEM model conducted over the pooled sample are shown in Fig. 1, controlling for the countries where the interviews were conducted. Higher evaluative (β = −0.35) and experienced well-being (β = −0.14) were associated with lower WHODAS scores. On the other hand, a higher age was associated with worse health (β = 0.35). The goodness-of-fit indices showed adequate values for this model: RMSEA = 0.079 [90% CI = (0.078, 0.080)]; SRMR = 0.043.

The SEM models conducted separately over each country (Table 3) showed a similar pattern in the association between evaluative well-being and health (with beta coefficients ranging from −0.56 in Ghana to −0.30 in China). Evaluative well-being was significantly associated (*p* < 0.001) with the WHODAS score in each country, while experienced well-being showed a significant relationship in five of the nine countries. In all countries, a higher age was strongly associated with worse health (with beta coefficients ranging from 0.11 in Finland to 0.41 in Russia). Regarding the remaining covariates, the results found across countries were similar to those found previously using the models conducted over the pooled sample.

**Table 3:** SEM estimates (95% CI) of the association of experienced and evaluative well-being with WHODAS score, in each country

	China	Finland	Ghana	India	Mexico	Poland	Russia	South Africa	Spain
Experienced well-being	−0.13*** (−0.19,−0.08)	−0.04 (−0.12,0.04)	−0.06 (−0.14,0.02)	−0.20*** (−0.24,−0.17)	−0.25*** (−0.38,−0.12)	−0.15*** (−0.20,−0.09)	−0.06 (−0.12,0.01)	−0.13* (−0.25,−0.01)	0.01 (−0.02,0.03)
Evaluative well-being	−0.30*** (−0.33,−0.27)	−0.50*** (−0.55,−0.44)	−0.56*** (−0.60,−0.52)	−0.40*** (−0.43,−0.37)	−0.41*** (−0.53,−0.30)	−0.37*** (−0.44,−0.30)	−0.41*** (−0.47,−0.36)	−0.37*** (−0.46,-0.29)	−0.43*** (−0.47,−0.39)
Sex (ref. Female)	−0.03** (−0.06,−0.01)	-0.01 (−0.05,0.03)	−0.06* (−0.11,−0.02)	−0.12*** (−0.15,−0.10)	−0.01 (−0.10,0.10)	−0.03 (−0.07,0.01)	−0.01 (−0.05,0.05)	−0.03 (−0.12,0.05)	−0.05** (−0.08,−0.02)
Age	0.34*** (0.30,0.38)	0.11*** (0.05,0.16)	0.23*** (0.18,0.28)	0.35*** (0.32,0.37)	0.31*** (0.20,0.42)	0.30*** (0.24,0.35)	0.41*** (0.35,0.46)	0.37*** (0.30,0.44)	0.29*** (0.25,0.33)
Married or in partnership (ref. No)	−0.14*** (−0.18,−0.10)	−0.04 (−0.08,0.01)	−0.03 (−0.07,0.01)	−0.05*** (−0.07,−0.03)	−0.05 (−0.13,0.03)	−0.07*** (−0.11,−0.03)	−0.08** (−0.13,−0.03)	−0.04 (−0.13,0.05)	−0.09*** (−0.12,−0.06)
Education attainment (ref. Lower than secondary school)	−0.05* (−0.08,−0.02)	−0.10*** (−0.15,−0.05)	−0.04* (−0.08,0.01)	−0.05*** (−0.08,−0.03)	−0.05 (−0.15,0.05)	−0.15*** (−0.20,−0.11)	−0.10*** (−0.16,-0.05)	−0.02 (−0.11,0.07)	−0.08*** (−0.11,−0.05)
RMSEA (90% CI)	0.030 (0.026,0.035)	0.102 (0.090,0.115)	0.024 (0.017,0.033)	0.062 (0.057,0.067)	0.031 (0.020,0.043)	0.059 (0.051,0.068)	0.032 (0.024,0.041)	0.039 (0.031,0.048)	0.069 (0.062,0.077)
SRMR	0.044	0.074	0.031	0.066	0.065	0.062	0.042	0.091	0.068

Beta (standardised) coefficients; *p < 0.05; **p < 0.01; ***p < 0.001

Weighted data

## Discussion

The present study analysed the association of the different components of well-being with health status in countries having different income levels. The two African countries, Ghana and South Africa, showed the best net affect (positive affect minus negative affect), whereas Mexico, Russia, and India experienced the worst affect, with the lowest scores for positive affect and high scores for negative affect. The rank for evaluative well-being differed substantially, and was similar to the rank found in other studies [39]. Finland was the country with the highest mean scores for evaluative well-being, followed by the two other high-income countries, and Russia was the country with the lowest score. This might be due to the fact that the emotions that people experience in their day-to-day lives are not so much determined by the income but other factors such as the strength of the social ties and the time they devote to stimulating and challenging activities, whereas the life satisfaction is more dependent on the income [40].

Regarding health status, China and Finland reported the best health status, and India and Russia the worst. The health ranking across countries was similar to the one found in the 2002 World Health Survey (WHS), in which of the nine countries analysed in the present study, Russia and India also obtained the lowest scores for self-reported health (measured from an overall health question), although South Africa and Ghana self-reported the best health on the WHS [41].

Although assumed, this is the first study that showed that well-being is associated with health, after controlling for potential confounders across countries with different income levels and that disentangled the differential association of each aspect of well-being with health. Evaluative well-being showed a stronger association with health than both the daily positive and negative emotions that people experience. Other studies have found that both experienced positive affect and evaluative well-being were associated with significantly reduced mortality [12, 13]. Furthermore, as found in previous studies, [42] the results of the present study showed that negative affect’s association with health was stronger than that of positive affect. The fact that other studies failed to demonstrate the association between positive affect and health [17] could be due to the instruments used to measure positive affect, which asks participants to report the symptoms experienced during the preceding week and therefore is more subject to memory and judgemental biases than the Day Reconstruction Method and other Experience Sampling Methods.

The association between evaluative well-being and health was significant in the nine countries analysed; notwithstanding, the association between experienced well-being and health was significant only in some countries, although all of them showed a trend towards this association. The different patterns observed across countries may be related to differences in the countries’ gross domestic product, social protection system, economic situation, health care provision, lifestyle behaviours, or living conditions.

The fact that evaluative well-being is more predictive of health than experienced well-being suggests that our level of satisfaction our lives might be more important for our health than the actual emotions than we experience in our day-to days lives.

The results of the present study need to be interpreted taking into account some limitations. The eudaimonic component of well-being was not evaluated. Since previous studies have found an association of purpose in life [43] and sense of coherence [44] with mortality, future studies should also include this component of well-being. The instrument used to assess evaluative well-being included questions about satisfaction with health, which could be highly correlated with health status. Nevertheless, when a general question about happiness was used as a measure of evaluative well-being (*Taking all things together, how would you say you are these days?*), evaluative well-being still had a higher impact than experienced well-being (results available upon request). Another caveat of the present study, as in all cross-sectional research, is that it is not possible to establish causality.

Several studies have previously shown a higher level of well-being in older people, in comparison with younger populations [45, 46]. As many other ageing studies, such as the Survey of Health, Ageing and Retirement in Europe (SHARE), the Health and Retirement Study (HRS) or the English Longitudinal Study on Ageing (ELSA), the SAGE and COURAGE in Europe projects are focused on the transition of ageing. Although the samples in the nine countries were nationally representative of the adult population in each country, people older than 80 years were overrepresented in the sampling in order to avoid having small sample sizes for the oldest age groups. It could be therefore possible that the high mean age in the sample in this study could influence the relationship between well-being and health. For this reason, all the statistical models considered in the present work have been adjusted for age (and other potential confounders).

## Conclusions

This multi-continent study fills a gap in knowledge by analysing the association of both the experiential and the evaluative components of well-being with health using the same methodology and the same evaluation instruments in such a large cohort of representative samples from nine countries with different income levels, medical resources, and at different stages of demographic and socio-economic evolution. The results of the present study provide additional information on the association of different components of well-being with health. Evaluative well-being showed a stronger association with health than experienced well-being, whereas negative affect showed a stronger association than positive affect. At the policy level, these results indicate that the strategies to improve health may not necessarily need to target health directly; indeed, it might be more efficient to invest in interventions that improve evaluative well-being and to encourage people to take such steps as engaging in leisure activities or reducing their commuting time in order to improve their health and well-being. This implies that the health sector could also work with other sectors, such as culture, infrastructure, and urban planning, to make changes that will improve population health. Future studies are needed that can infer causality from these associations, as well as studies to evaluate the eudaimonic component of well-being.
